# Supplementary material for: Evaluating the reliability, validity, and utility of overlapping networks: Implications for network theories of cognition
Source: Hum Brain Mapp. 2022 Nov 1;44(3):1030–45. doi: 10.1002/hbm.26134 (PMC9875920; doi:10.1002/hbm.26134)
Supplement: Supplementary file 1 — Figure S1 Distribution of assignment weights for regions assigned to each overlapping network. Histograms are restricted to ROIs with an assignment weight greater than zero for each network and presented with 10 equally spaced bins from 0 to 1. [file HBM-44-1030-s001.docx]

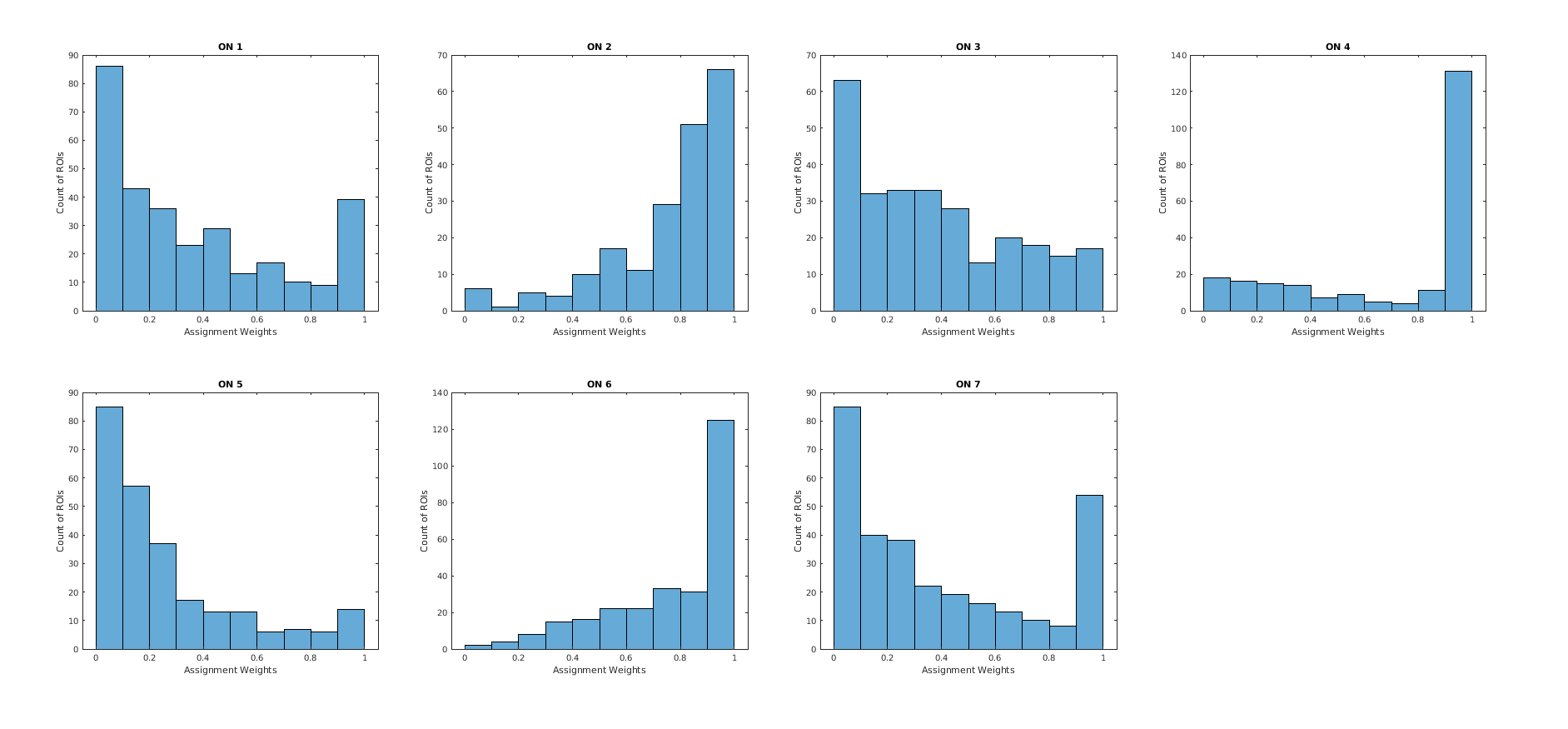


**Supplementary Figure 1. Distribution of assignment weights for regions assigned to each overlapping network.** Histograms are restricted to ROIs with an assignment weight greater than zero for each network and presented with 10 equally spaced bins from 0 to 1.
