# Supplementary material for: Evaluating the reliability, validity, and utility of overlapping networks: Implications for network theories of cognition
Source: Hum Brain Mapp. 2022 Nov 1;44(3):1030–45. doi: 10.1002/hbm.26134 (PMC9875920; doi:10.1002/hbm.26134)
Supplement: Supplementary file 2 — Figure S2 Framewise displacement of Exploratory and Confirmatory samples. Framewise displacement was calculated as the sum of the absolute value of the differences in the first six motion parameters (3 translations, 3 rotations) between the current frame and the preceding frame. Rotation parameters were converted to millimeters by calculating the arc length of the angle for a sphere of 50 mm to approximate the distance of the cortex from the center of the head. Procedures were replicated from those reported by Power et al. (2012). Blue lines for each sample indicate the average framewise displacement across all scans and timepoints. [file HBM-44-1030-s002.docx]

**
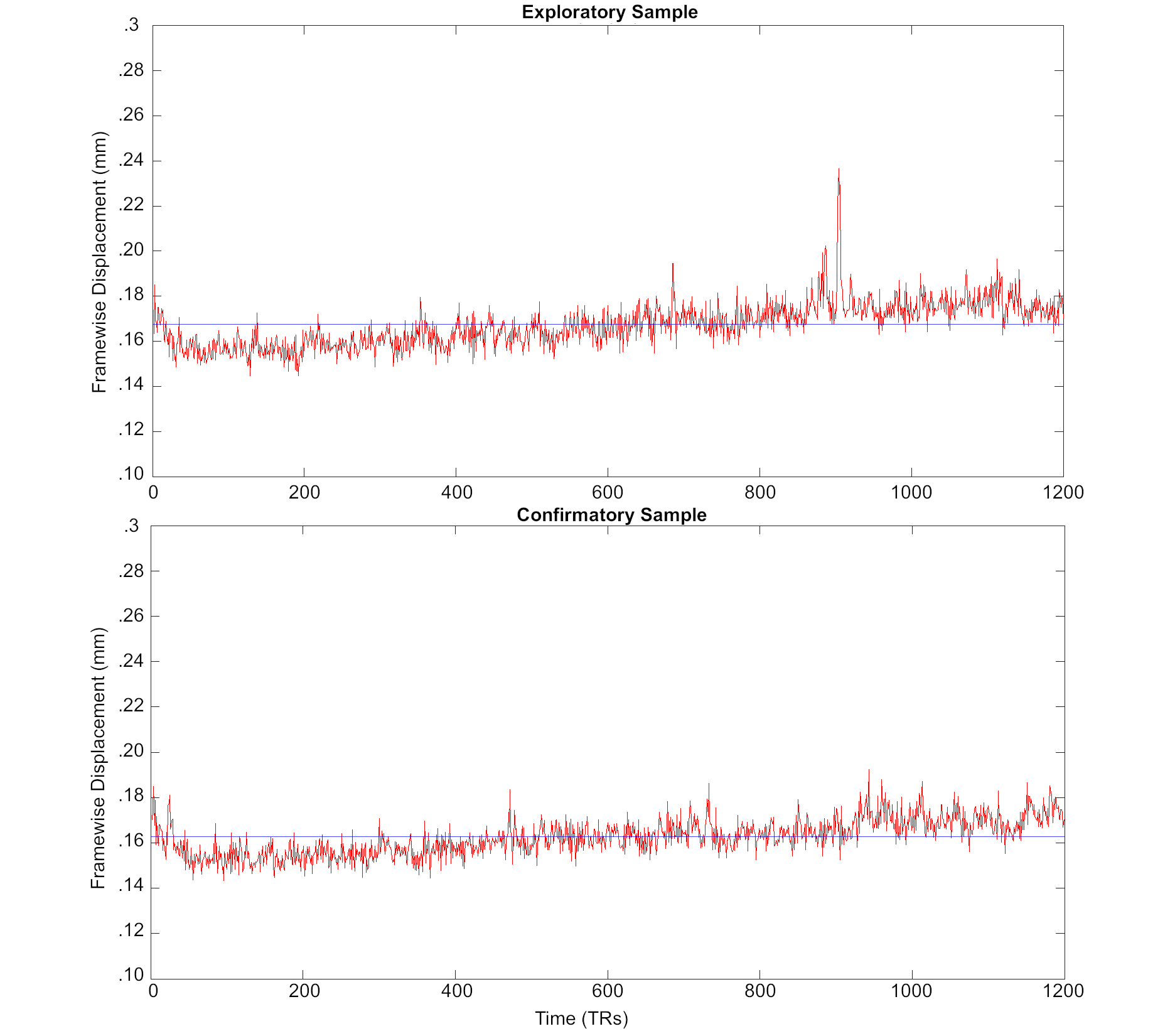
**

**Supplementary Figure 2. Framewise displacement of Exploratory and Confirmatory samples.** Framewise displacement was calculated as the sum of the absolute value of the differences in the first six motion parameters (3 translation, 3 rotation) between the current frame and the preceding frame. Rotation parameters were converted to millimeters by calculating the arc length of the angle for a sphere of 50mm to approximate the distance of the cortex from the center of the head. Procedures were replicated from those reported by Power and colleagues (2012). Blue lines for each sample indicate the average framewise displacement across all scans and timepoints.
